# Species, Risk Factors, and Antimicrobial Susceptibility Profiles of Bacterial Isolates from HIV-Infected Patients Suspected to Have Pneumonia in Mekelle Zone, Tigray, Northern Ethiopia

**DOI:** 10.1155/2019/8768439

**Published:** 2019-05-05

**Authors:** Gebre Adhanom, Dawit Gebreegziabiher, Yemane Weldu, Araya Gebreyesus Wasihun, Tadele Araya, Haftom Legese, Bruno S. Lopes, Muthupandian Saravanan

**Affiliations:** ^1^Department of Medical Microbiology and Immunology, Division of Biomedical Sciences, School of Medicine, College of Health Science, Mekelle University, P.O. Box 1871, Mekelle, Ethiopia; ^2^Department of Medical Laboratory Science, College of Medicine and Health Science, Adigrat University, Ethiopia; ^3^Department of Medical Microbiology, School of Medicine, Medical Sciences and Nutrition, University of Aberdeen, 0:025 Polwarth Building, Aberdeen AB25 2ZD, UK

## Abstract

**Background:**

Pneumonia is a condition, where bacterial infections are implicated as the most common causes of morbidity and mortality in humans. The actual burden of HIV-infected patients with pneumonia is not well documented in Mekelle region of Ethiopia. This study estimated the prevalence of bacterial pneumonia in HIV patients, antimicrobial susceptibility patterns of pathogens implicated in pneumonia, and associated risk factors in Mekelle zone, Tigray, Northern Ethiopia, during August-December 2016.

**Methods:**

Sputum specimens were collected from 252 HIV seropositive individuals with suspected pneumonia. Data on sociodemographics and risk factors were also collected using a structured questionnaire. Blood, Chocolate, and Mac Conkey agar plates (Oxoid, Hampshire, UK) were used to grow the isolates. The isolated colonies were identified based on Gram stain, colony morphology, pigmentation, hemolysis, and biochemical tests. The antimicrobial susceptibility test was performed using the modified Kirby-Bauer disc diffusion method. The analysis was performed using SPSS version 22 and p-value < 0.05 with corresponding 95% confidence interval (CI) was considered statistically significant.

**Results:**

Out of the 252 samples, 110 (43.7%) were positive for various bacterial species. The predominant bacterial species were* Klebsiella pneumoniae* (n=26, 23.6 %) followed by* Streptococcus pneumoniae* (n=17, 15.5 %),* Escherichia coli* (n=16, 14.5%),* Klebsiella *spp. (n=15, 13.6%)*, Staphylococcus aureus *(n=9, 8.2%),* Enterobacter* spp. (n=7, 6.3%),* Pseudomonas aeruginosa *(4, n=3.6%),* Proteus* spp. (n=4, 3.6%),* Citrobacter freundii* (n=7, 6.3%),* Streptococcus pyogenes *(3, 2.7%), and* Haemophilus influenzae *(n=2, 1.8%). Young age (18-29), recent CD4^+^ count less than 350 cells/mL, alcohol consumption, and HIV WHO stage II showed significant association with the occurrence of bacterial pneumonia. Resistance to penicillin, co-trimoxazole, and tetracycline was observed in 81.8%, 39.8%, and 24.5% of the isolates, respectively.

**Conclusions:**

The problem of pneumonia among HIV patients was significant in the study area. The high prevalence of drug-resistant bacteria isolated from the patient's samples possesses a health risk in immunocompromised HIV patients. There is a need to strengthen and expand culture and susceptibility procedures for the administration of appropriate therapy to improve patients management and care which may aid in decreasing the mortality.

## 1. Background

Respiratory infections are the leading cause of morbidity and mortality in HIV-infected individuals [1]. It is also the most frequent cause of hospital admission in HIV-infected people worldwide with an incidence of 20-25 episodes per 100 hospital admissions per year [2]. About 70% of HIV patients have a pulmonary complication during the evolution of the disease, mainly as a consequence of infectious etiology [2]. During respiration, the respiratory tract, especially nose, nasopharynx, and oropharynx, comes into contact with a large number of bacteria that may cause life-threatening respiratory infections [3]. Lower respiratory tract infections (LRTIs) are among the most prevalent diseases of human beings worldwide [4], which result in around 7 million deaths annually [5]. Pneumonia is an acute respiratory illness which is one of the most common LRTI, mainly caused by bacteria, viruses, and fungi [6, 7].

Bacterial pneumonia is the most frequent cause of pulmonary infections, which is among the most common causes of respiratory failure in HIV- infected patients [2, 8]. The incidence of bacterial pneumonia in HIV-infected patients ranges from 1.93 to 19.2% of cases per year in Nigeria [9]. HIV-infected individuals have a sixfold greater risk of bacterial pneumonia than the noninfected individuals [10] and estimated greater than one-third of all persons with AIDS will develop at least one episode of severe bacterial pneumonia over the course of their HIV infection [10]. If bacterial pneumonia is not properly treated, it causes further complications leading to empyema, meningitis, pericarditis, hepatitis, and arthritis [10].

The most common pathogens that cause bacterial pneumonia are* S. pneumoniae*,* H. influenzae*,* K. pneumoniae*,* P. aeruginosa*,* E. coli*, and* S. aureus *[11]. Among this* S. pneumoniae* is considered the predominant bacterial pathogen across all age groups and accounts for approximately 30% of pneumonia cases [12].

The incidence of bacterial drug resistance is high due to empirical drug administration and poor adherence to treatment standards. Hence, specific antibiotic therapy is fundamental to manage and control bacterial pneumonia in HIV patients [13]. Therefore, identifying the bacterial cause and performing antibiotic susceptibility testing are vital [2].

In Northern Ethiopia, there is a high prevalence of HIV-infected patients [14]. However, there is no study which shows the real burden of bacterial pneumonia among HIV positive individuals in this region. In addition, the antimicrobial susceptibility patterns of the common bacterial agents for pneumonia are not known. Therefore, this study was performed to assess the burden of bacterial pneumonia, the risk factors, and antimicrobial susceptibility of bacterial isolates in HIV positive individuals with suspected pneumonia at ART clinics of Mekelle zone, Tigray, Northern Ethiopia.

## 2. Methods

### 2.1. Study Design, Specimen Collection, and Transportation

A multicentre cross-sectional study was conducted from August-December 2016 at different ART clinics of Mekelle zone, Tigray, Ethiopia. A total of 252 HIV patients with suspected pneumonia were included in the study based on the inclusion/exclusion criteria. Sociodemographic, clinical, and risk-factor related data were collected using a structured and pretested questionnaire. Pneumonia suspected individuals showing clinical symptoms such as shortness of breath, chest pain, fever, chills, tiredness, and cough were examined by clinicians and the sputum samples were collected and transported to medical microbiology laboratory, Mekelle University, College of Health Sciences by medical laboratory technologists for analysis within 2 hrs or stored at 4°C until further processing.

### 2.2. Inclusion and Exclusion Criteria

HIV seropositive, highly active antiretroviral therapy users and nonusers aged ≥ 18 with a diagnosis of pneumonia as described by the clinician were included. Patients who were critically ill, who cannot produce sputum, suspected pulmonary tuberculosis and who took antibiotics during the past two week period were excluded.

### 2.3. Specimen Processing

Smear was prepared from each sputum sample for Gram staining and slides were examined under low power field (l0X). Sputum samples with > 25 polymorphonuclear leukocytes and <10 epithelial cells were accepted for culture. Samples that had more than 10 epithelial cells and less than 25 polymorphonuclear leukocytes per low power field were discarded [15, 16].

### 2.4. Cultivation and Identification of Isolates

Blood, Chocolate, and Mac Conkey agar plates (Oxoid, Hampshire, UK) were prepared as per the manufacturers' instructions. Using a calibrated wire loop, approximately 0.1*μ*L purulent sputum sample was streaked onto each agar plate. Chocolate agar plates were incubated at 37°C for 24 hours in a candle jar. MacConkey agar and blood agar plates were incubated aerobically at 37°C for 24 hours [15, 16]. After incubation, the plates were inspected for any growth and negative plates were incubated for an additional 24 hours. Quantitative sputum culture analysis was carried out and the significant bacterial count was reported on observing an excess of 10^6^ CFU/mL [17]. More than 100 CFU were considered significant for bacterial growth in this study. A single pure culture colony of each isolate was stored in nutrient broth with 10% glycerol stock solution for identification. Gram-positive cocci were differentiated and identified based on Gram stain, patterns of hemolysis on blood agar, colonial characteristics, catalase test, coagulase test, optochin susceptibility, and bacitracin susceptibility. Gram-negative bacteria were also identified based on Gram stain, colonial morphology and pigmentation, oxidase test, carbohydrate fermentation, H_2_S production, citrate utilization, motility, indole formation, urea hydrolysis and growth in blood agar with “X”, and “V” factors for* H. influenzae*.

### 2.5. Antimicrobial Susceptibility Testing

The antimicrobial susceptibility test was performed using the Kirby-Bauer disc diffusion test method [16]. The antimicrobial discs used for susceptibility testing were amikacin (AK,30***μ***g), ciprofloxacin (CIP,5***μ***g), ceftriaxone (CTR,30***μ***g), chloramphenicol (C,10***μ***g),co-trimoxazole (COT,25***μ***g), erythromycin (ERY,15***μ***g), gentamicin (GN,10***μ***g), Methicillin (MET, 30***μ***g)penicillin (P,10 units), and tetracycline (TE,30***μ***g). Muller-Hinton agar (MHA) plates were used for the nonfastidious organism, whereas MHA supplemented with 5% sheep blood were used for* S. pneumoniae *and* Haemophilus* spp. using a standard procedure [16]. The results were interpreted using the guidelines described by the CLSI (2016) standards. Multidrug resistance was defined by nonsusceptibility to at least one agent in three or more antimicrobial categories as described earlier by Magiorakos* et al.* [18].

### 2.6. Quality Assurance

The training was provided to data collectors on how to perform the questionnaire and sampling processing. The filled questionnaires were checked for their completeness. Media preparations were made based on the manufacturer's instruction. Quality control slides were incorporated during Gram staining. Standard operating procedures were followed during specimen collection, handling, transportation, microbiological analysis, and interpretation. Five percent of media per batch were incubated overnight for sterility check. Reference strains* S. aureus *ATCC25923,* E. coli* ATCC25922, and* P. aeruginosa *ATCC27853 were used during cultivation and antimicrobial susceptibility testing [16].

### 2.7. Statistical Analysis

Data was analyzed using SPSS version 22, and descriptive statistics, bivariate, and multivariate logistic regression were employed. A bivariate logistic regression model was used to determine the possible association of each variable with the outcome variable and multivariate analysis was done to those independent variables with p-value < 0.05 to identify factors that are independently associated with the dependent variable. P-value <0.05 with 95% confidence interval was considered as statistically significant.

## 3. Results

### 3.1. Sociodemographic Characteristics

This study was conducted among 252 study participants. Out of 224 (88.9%) were urban dwellers and 28 (11.1%) were rural dwellers. The mean age of study participants was 40.58 ± 9.9, range 18-67 years old, and 50% being male and the rest female (Table 1).

### 3.2. Prevalence of Bacterial Pneumonia

Bacterial pneumonia was observed in 110 (43.7 %) and out of those 59 (46.6 %) of them were male participants (Table 2). Bacterial pneumonia was found highest in age group 18-29 (n=15, 50 %) and lowest in the age group 50-67, 14 (28.6%). Out of the 110 isolates, 95 (42.4 %) were isolated from urban dwellers. The predominant bacterial species were* Klebsiella pneumoniae* (n=26, 23.6 %) followed by* Streptococcus pneumoniae* (n=17, 15.5 %),* Escherichia coli* (n=16, 14.5%),* Klebsiella *spp. (n=15, 13.6%)*, Staphylococcus aureus *(n=9, 8.2%),* Enterobacter* spp. (n=7, 6.3%),* Pseudomonas aeruginosa *(4, n=3.6%),* Proteus* spp. (n=4, 3.6%),* Citrobacter freundii* (n=7, 6.3%),* Streptococcus pyogenes *(3, 2.7%), and* Haemophilus influenzae *(n=2, 1.8%).

### 3.3. Antimicrobial Susceptibility Pattern of Bacterial Isolates

Out of the tested antibiotics, 39.8% (n=40 isolates) were resistant to co-trimoxazole and 24.5% (n=26) for tetracycline. In this study, 81.8% (n=9) Gram-positive isolates and 27.6% (n=8) were resistant to penicillin and erythromycin, respectively (Table 3). Similarly, 10.7% (n=8) of Gram-negative isolates were resistant to ceftriaxone (Table 3). On the other hand, most of the isolates were less resistant to amikacin (n=6, 6.8%) and ciprofloxacin (n=8, 9.1%). Methicillin resistance was observed in 44.4% (n=4) isolates of* S. aureus *(Table 3). Multidrug resistance was observed in 18% (n=15) of isolates (Table 4).

### 3.4. Associated Risk Factors

All variables with a significance value in the bivariate analysis were entered in to multiple logistic regression model and in multivariate analysis the variables age groups 18-29 (AOR = 3.8, 95 % CI: 1.25-11.52, P ≤ 0.018), age groups 30-39 (AOR = 3.4, 95% CI: 1.37-8.25, P ≤ 0.008), recent number of CD4+ cell count less than 350 (AOR = 2.4, 95 % CI:1.11-5.09, P≤ 0.026), alcohol consumption (AOR= 7.4, 95 % CI: 2.46-22.11, P ≤ 0.001), and WHO stage of II HIV infection (AOR= 5.4, 95 % CI: 2.00-14.36, P ≤ 0.001) were found to have statistically significant association with bacterial pneumonia. In the bivariate analysis, gender and occupation failed to show a significant association with the occurrence of bacterial pneumonia. Although not statistically significant, bacterial pneumonia was higher in cigarette smokers, in non-cART users, and in those who interrupt cART (Table 5).

## 4. Discussion

In this study, the overall prevalence of bacterial pneumonia was found to be 43.7%. This is in line with the studies reported from Nigeria 42.9 % [19] and India 44.3 % [20]. This is due to the fact that individuals with compromised immune status are more susceptible to bacterial infections. On the other hand, our finding is higher than the findings from Malawi 29 % [13] and India 18.4 % [21] and lower than the study reported from Nigeria 55.6% [9]. The inconsistency may be due to sampling size variation, type of sample used, and immune status of the study participants.

In our study,* K. pneumoniae *and* S. pneumoniae *were the predominant pathogens isolated as reported in similar studies from Nepal [22, 23] and India [20]. On the other hand, different predominant isolates, such as* E. coli* and* P. aeruginosa* were reported from Nigeria [9] while* S. pneumoniae *and* H. influenzae *were reported from Uganda as dominant bacterial species [24]. A climatic and geographic variation could be the possible reasons for the different frequency of the bacterial isolates in different study area among HIV patients.

*E. coli *was the second most common Gram-negative bacterium to be isolated in our study; which in agreement with the study reported from Bahrain [25] and Nigeria [9]. Among the Gram-positives* S. aureus* was the second most common species to be isolated; this is consistent with a study reported from the United States [26], Italy [27], and Greece [28]. The occurrence might be due to aspiration of the bacteria from the previously colonized site, prior hospitalization, contaminated foods eaten, and mostly systemic spread from blood to the lungs in these patients [26–28].

WHO recommends co-trimoxazole as an intervention to reduce infections by opportunistic pathogens including bacterial pneumonia in HIV positive patients. In this study, about 39.8% and 24.5% of bacterial isolates were resistant to co-trimoxazole and tetracycline. This is similar to the study reported in Nigeria [9] and Tanzania [20]. This may be due to the consistent use of this antibiotic, which might contribute to the development of drug resistance. Out of the tested Gram-positive isolates, 81.8 % and 27.6 % were resistant to penicillin and erythromycin respectively. This might be because these antibiotics have been very commonly prescribed for many years during the course of infection.

According to the international standard for the definition of drug resistance [18], 17.9% isolates were multidrug resistant. We did not have data from our study participants engaging in self-medication but empirical treatment still is a contributing factor for the emergence of drug resistance in these patients [9, 29]. Out of the* S. aureus *isolates, 44.4 % were methicillin resistance but because the sample size was low these results may not be significant. This is higher from the study reported from Bahrain, 10.5 % [25], and lower from Italy, 65% [27]

In this study, age groups 18-29 and 30-39, alcohol consumption, recent CD4^+^ count less than 350 cells/***μ***L, and WHO stage II of HIV infection showed significant association to have bacterial pneumonia. Age groups, 18-29 and 30-39, were 3.8 and 3.4 times more likely to have bacterial pneumonia, respectively, compared to the age group, 50-67 which is in agreement with studies reported from Nigeria [9] and Nepal [22]. The reason may be due to the fact that a large number of younger age groups of our study participants were within CD4^+^ cell count less than 350 cells/***μ***L and WHO stage II compared to those aged between 50 and 67. This can contribute to increased bacterial pneumonia observed in our study participants. On the contrary, the studies reported in Spain [2], Netherlands [30], and France [31] bacterial pneumonia is also common in older age groups. The immune status, place of living, diet, deprivation, and affluence are some of the factors which may play a role in influencing the level of illness in a given population.

Alcohol consumption is one of the potential risk factors identified, though our study did not identify the level of consumption; alcohol consumers were 7.4 times more likely to have bacterial pneumonia [32]. This may be due to the fact that alcohol consumers are prone to more oropharyngeal secretions due to alteration of their level of consciousness. Alcohol also has an effect on depressing cough, decreasing endothelial adherence, lowering chemotaxis, suppressing B and T-cell expansion, and function which contributes to poor clearance mechanism of lung cells which is in line with the studies reported from South Africa [32], Europe [33], and western North America [34].

In this study, recently low CD4^+^ cell count was the other risk factor identified and individuals with recent CD4^+^ cell count less than 350 cells/***μ***L were 2.4 times more likely to have bacterial pneumonia compared to those who had CD4^+^cell counts ≥ 500 cells/***μ***L, consistent with the study reported from United States [10] and France [31]. WHO stage II of HIV infection is the other risk factor and these individuals were 5.4 times more likely to have bacterial pneumonia compared to those WHO stage I individuals. In addition to this, even though WHO stage III was not significantly associated, the probability of getting bacterial pneumonia is still higher in these patients comparing to WHO stage I. This might be related to unprogrammed activation of the immune system due to recurrent infections which contribute to damage of immune cells and the physical barriers of our respiratory mucosal membrane and simply make susceptible to different opportunistic infections [6].

In this study, although not statistically significant, bacterial pneumonia was higher among cigarette smokers. Smoking lowers the action and number of cilia and inhibits the ability to prevent the entry of microorganisms to the respiratory tract. This is supported by studies reported earlier which indicate cigarette smoking increases the risk of bacterial pneumonia two times compared to nonsmokers [35].

## 5. Conclusions

In this study, the prevalence of bacterial pneumonia was 43.7 % caused majorly by Gram-negative bacterial species such as* K. pneumoniae* and* E. coli*. In Gram-positive isolates,* S. pneumoniae* and* S. aureus *were the most dominant species of bacteria. Most isolates were susceptible to amikacin, ciprofloxacin, and ceftriaxone. Younger age group, the recent number of CD4+ cell count less than 350 cells, alcohol consumption, and WHO stage II of HIV infection showed significant association with bacterial pneumonia. Identification of the etiological agent and performing antimicrobial susceptibility testing in order to select an appropriate antimicrobial agent will be important in the management of bacterial pneumonia and limiting the evolution of resistant bacteria in the clinical setting.

## Figures and Tables

**Table 1:** Sociodemographic characteristics of the study participants in ART clinics of Mekelle zone, Tigray, Northern Ethiopia, August 2016-December 2016 (n=252).

Variables	Frequency	Percent (%)

Gender		
Male	127	50.4
Female	125	49.6
Age (in years)		
18-29	30	11.9
30-39	98	38.9
40-49	75	29.8
50-67	49	19.4
Residence		
Urban	224	88.9
Rural	28	11.1
Occupation		
Farmer	19	7.5
Employed	78	31
Unemployed	110	43.6
Self-employed	45	17.9
Educational level		
Illiterate	42	16.7
Read and write	33	13.1
1-8	80	31.7
9-12	59	23.4
College and above	38	15.1

**Table 2:** Rate of bacterial pneumonia with regard to the sociodemographic characteristics among pneumonia suspected HIV patients in ART clinics of Mekelle zone, Northern Ethiopia, August 2016-December 2016.

Variables	Bacterial occurrence		Total
	Positive N (%)	Negative N (%)	

Gender			
Male	59 (46.6)	68 (53.5)	127
Female	51 (40.8)	74(59.2)	125
Age (in years)			
18-29	15 (50)	15 (50)	30
30-39	47 (48)	51 (52)	98
40-49	34 (45.3)	41 (54.7)	75
50-67	14 (28.6)	35 (71.4)	49
Residence			
Urban	95 (42.4)	129 (57.6)	224
Rural	15 (53.6)	13 (46.4)	28
Occupation			
Farmer	9 (47.4)	10 (52.6)	19
Employed	32 (41)	46 (59)	78
Unemployed	46 (41.8)	64 (58.2)	110
Self-employed	23 (51.1)	22 (48.9)	45
Educational level			
Illiterate	14 (33.3)	28 (66.7)	42
Read and write	10 (30.3)	23 (69.7)	33
1-8	37 (46.3)	43 (53.7)	80
9-12	30 (50.8)	29 (49.2)	59
College and above	19 (50)	19 (50)	38

**Table 3:** Antimicrobial susceptibility patterns of bacterial isolates among pneumonia suspected HIV patients in ART clinics of Mekelle zone, Northern Ethiopia, August-December 2016.

Isolates(n)		Antibiotics									
		GN	AK	CTR	P	CIP	COT	ERY	C	TE	MET

*S. pneumoniae* (17)	S	NT	NT	NT	NT	NT	6(35.3)	11(64.7)	14(82.4)	10(58.8)	NT
	R						11(64.7)	6(35.3)	3(17.6)	7(41.2)	
*S. aureus *(9)	S	8(88.9)	9(100)	NT	2(22.2)	8(88.9)	6(66.7)	7(77.8)	9(1000	7(77.8)	5(55.6)
	R	1(11.1)	0		7(77.8)	1(11.1)	3(33.3)	2(22.2)	0	2(22.2)	4(44.4)
*S. pyogenes *(3)	S	NT	NT	NT	1(33.3)	NT	3(100)	NT	1(33.3)	0	NT
	R				2(66.7)		0		2(66.7)	3(100)	
*K. pneumoniae *(26)	S	21(80.8)	23(88.5)	24(92.3)	NT	26(100)	15(57.7)	NT	23(88.5)	19(73.1)	NT
	R	5(19.2)	3(11.5)	2(7.7)		0	11(42.3)		3(11.5)	7(26.9)	
*E. coli *(16)	S	14(87.5)	15(93.8)	13(81.3)	NT	14(87.5)	10(62.5)	NT	16(100)	15(93.8)	NT
	R	2(12.5)	1(6.2)	3(18.7)		2(12.5)	6(37.5)		0	1(6.2)	
*Klebsiella *spp. (15)	S	14(93.3)	14(93.3)	14(93.3)	NT	14(93.3)	14(93.3)	NT	15(100)	14(93.3)	NT
	R	1(6.7)	1(6.7)	1(6.7)		1(6.7)	1(6.7)		0	1(6.7)	
*Enterobacter *spp.(7)	S	7(100)	7(100)	7(100)	NT	3(42.9)	3(42.9)	NT	3(42.9)	7(100)	NT
	R	0	0	0		4(57.1)	4(57.1)		4(57.1)	0	
*Proteus *spp.(4)	S	4(100)	3(75)	4(100)	NT	4(100)	4(100)	NT	3(75)	3(75)	NT
	R	0	1(25)	0		0	0		1(25)	1(25)	
*Citrobacter freundii *(7)	S	7(100)	7(100)	5(71.4)	NT	7(100)	5(71.4)	NT	5(71.4)	5(71.4)	NT
	R	0	0	2(28.6)		0	2(28.6)		2(28.6)	2(28.6)	
*P. aeruginosa *(4)	S	3(75)	4(100)	NT	NT	4(100)	NT	NT	NT	NT	NT
	R	1(25)	0			0					
*H. influenzae *(2)	S	NT	NT	NT	NT	NT	0	NT	2(100)	0	NT
	R						2(100)		0	2(100)	
Total n (%)	S	78(88.6)	82(93.2)	67(89.3)	3(27.2)	80(90.9)	62(60.8)	21(72.4)	91(85.8)	80(75.5)	5(55.6)
	R	10(11.4)	6(6.8)	8(10.7)	9(81.8)	8(9.1)	40(39.8)	8(27.6)	15(14.2)	26(24.5)	4(44.4)

NT= not tested, *Klebsiella spp. = *other *Klebsiella species. *P = penicillin, CTR= ceftriaxone, GN = gentamicin, AK= amikacin, COT= co-trimoxazole, TE = tetracycline, C = chloramphenicol, CIP = ciprofloxacin, MET= methicillin, ERY= erythromycin, S= susceptible, and R= resistant. % is computed from each cell. Total= total calculated from both Gram negative and positive isolates for that specific antibiotics were tested.

**Table 4:** Multidrug resistance patterns of bacterial pneumonia from pneumonia suspected HIV patients in ART clinics of Mekelle zone, Northern Ethiopia, August-December 2016.

Bacterial Isolates	Antibiotics						Total
	Ro	R1	R2	R3	≥ R4	MDR	

K. pneumoniae	9(34.6)	7(30)	8(30.8)	1(3.8)	1(3.8)	2(7.7)	26(100)
E. coli	9(56.3)	3(18.7)	2(12.5)	0(.00)	2(12.5)	2(12.5)	16(100)
Klebsiella spp.	13(86.6)	1(6.7)	0(.00)	0(.00)	1(6.7)	1(6.7)	15(100)
Citrobacter freundii	4(57.1)	1(14.3)	0(.00)	1(14.3)	1(14.3)	2(28.6)	7(100)
Enterobacter spp.	3(42.9)	0(.00)	0(.00)	4(57.1)	0(.00)	4(57.1)	7(100)
Proteus spp.	2(50)	1(25)	1(25)	0(.00)	0(.00)	0(.00)	4(100)
P. aeruginosa	3(75)	1(25)	0(.00)	0(.00)	0(.00)	NA	4(100)
H. influenzae	0(.00)	2(100)	0(.00)	0(.00)	0(.00)	NA	2(100)
S. pneumoniae	2(11.8)	4(23.5)	10(58.8)	1(5.9)	0(.00)	NA	17(100)
S. aureus	3(33.4)	1(11.1)	2(22.2)	1(11.1)	2(22.2)	4(44.4)^a^	9(100)
S. pyogenes	0(.00)	0(.00)	2(66.7)	1(33.3)	0(.00)	NA	3(100)

Total	48(43.6)	21(19.1)	25(22.7)	9(8.2)	7(6.4)	15(17.9)^b^	110(100)

R0: susceptible to all, R1: resistance to one, R2: resistance to two, R3: resistance to three, and ≥ R4: resistance to four and more antibiotics. MDR= multidrug resistance. ^a^Percent is computed from the total number of *S. aureus;*^b^percent is computed from a total number of isolates, based on which MDR definition is applied.

**Table 5:** Bivariate and multivariate logistic regression analysis of factors associated with bacterial pneumonia in pneumonia suspected HIV patients in ART clinics of Mekelle zone, Northern Ethiopia, August-December 2016.

Variables	Bacterial pneumonia		COR (95% CI)	P-value	AOR (95% CI)	P-value
	Positive	Negative				
	N (%)	N (%)				

Gender						
Male	59 (46.6)	68 (53.5)	1.3 (0.76-2.07)	0.366		
Female	51 (40.8)	74 (59.2)	1			
Age (in years)						
18-29	15 (50)	15 (50)	2.5 (0.97-6.44) ∗	0.058	3.8 (1.25-11.52) ∗	0.018
30-39	47 (48)	51 (52)	2.3 (1.10-4.81) ∗	0.026	3.4 (1.37-8.25) ∗	0.008
40-49	34 (45.3)	41 (54.7)	2.1 (0.96-4.47)	0.063	2.0 (0.81-5.06)	0.132
50-67	14 (28.6)	35 (71.4)	1		1	
Occupation						
Employed	32 (41)	46 (59)	1			
Farmer	9 (47.4)	10 (52.6)	1.1 (0.35-3.72)	0.825		
Unemployed	46 (40)	64 (60)	0.9 (0.30-2.66)	0.849		
Self-employed	23 (51.1)	22 (48.9)	0.6 (0.31-1.35)	0.248		
Recent CD4+ cells/*μ*l						
<350	56 (57.7)	41 (42.3)	3.3 (1.7-6.34) ∗	0.001	2.4 (1.11-5.09)∗	0.026
350-500	34 (39.1)	53 (60.9)	1.5 (0.78-3.03)	0.211	1.2 (0.54-2.58)	0.686
>500	20 (29.4)	48 (70.6)	1		1	
Cigarette smoking						
Yes	13 (65)	7 (35)	2.6 (0.99-6.72)	0.051	1.6 (0.44-5.50)	0.488
No	97 (41.8)	135 (58.2)	1		1	
Use cART						
Yes	100 (41.8)	139 (58.2)	1		1	
No	10 (76.9)	3 (23.1)	4.6 (1.24-17.27)	0.022	0.00	1.000
Ever interrupt cART						
Yes	16 (69.6)	7 (30.4)	3.6 (1.43-9.17) ∗	0.007	2.5 (0.87-7.23)	0.090
No	84 (38.7)	133 (61.3)	1		1	
Alcohol consumption						
Yes	22 (81.5)	5 (18.5)	6.9 (2.5-18.76) ∗	0.001	7.4 (2.46-22.11)∗	0.001
No	88 (39.1)	137 (60.9)	1		1	
WHO Stage						
I	80 (38.6)	127 (61.4)	1		1	
II	22 (75.9)	7 (24.1)	5.0 (2.04-12.22)∗	0.001	5.4 (2.00-14.36)∗	0.001
III	8 (50)	8 (50)	1.6 (0.57-4.40)	0.374	0.5 (0.13-1.97)	0.329

COR=crude odds ratio; CI=confidence interval; AOR=adjusted odds ratio; 1: referent; ∗statically significant.

## Data Availability

Data supporting the conclusions of this article are available by request from G. Adhanom. The relevant raw data will be made available to researchers wishing to use them for noncommercial purposes.

## Abbreviations

AIDS: Acquired Immune-Deficiency Syndrome
AOR: Adjusted Odds Ratio
ART: Antiretroviral Therapy
ATCC: American Type Culture Collection
CI: Confidence interval
CLSI: Clinical and Laboratory Standards Institute
COR: Crude Odds Ratio
HIV: Human Immunodeficiency Virus
MDR: Multidrug Resistance
SPSS: Statistical Package for Social Sciences.
